# Underestimating the impact of erect abdominal radiographs?

**DOI:** 10.1002/jmrs.334

**Published:** 2019-04-10

**Authors:** Kevin Flintham, Beverly Snaith

**Affiliations:** ^1^ Mid Yorkshire Hospitals NHS Trust Wakefield UK; ^2^ University of Bradford Bradford UK

We read with interest the editorial by Chawla and Peh^1^ regarding the use of abdominal radiographs in the emergency department. As the authors stated, the erect radiograph is not justified under UK guidance^2^ and was phased out a number of years ago. However, a recent information request confirmed that the erect abdominal radiograph is still employed across the globe.^3^

The statement from the authors that the inclusion of an erect abdominal radiograph, in addition to the supine projection, will double the radiation dose (0.7–1.4 mSv) does require challenging. Physical measurement data obtained from 168 patients collected for the SEPRAIDD project^4^ demonstrated differences in patient body habitus between a supine and standing posture. Importantly, the antero‐posterior soft tissue thickness increased by an average of 22% at the level of the iliac crests. This change is as a result of the removal of compressive forces and the effect of gravity, as well as organ repositioning.^5^ As most abdominal projections will be obtained with an automatic exposure control, the impact of the increased abdominal soft tissue on effective and absorbed dose will be significant.

We recognise the desire to reduce the number of abdominal radiographs performed in the acute setting, but they still have value to the clinician^6^, ^7^ in the absence of easy access to computed tomography (CT) or ultrasound scans. However, the erect radiograph provides little diagnostic benefit and we believe the radiation burden of this projection is significantly underestimated as the impact of postural changes on body habitus is poorly understood within the imaging community.

## Conflict of Interest

The authors declare no conflict of interest.
